# Expression of recombinant human lysozyme in transgenic chicken promotes the growth of *Bifidobacterium* in the intestine and improves postnatal growth of chicken

**DOI:** 10.1186/s13568-016-0280-2

**Published:** 2016-11-10

**Authors:** Hai Wang, Hongping Wu, Kejun Wang, Zhichen Cao, Kun Yu, Ling Lian, Zhengxing Lian

**Affiliations:** Beijing Key Laboratory of Animal Genetic Improvement, China Agricultural University, No.2 Yuanmingyuan West Rd, Haidian, Beijing, 100193 China

**Keywords:** rhLZ, Transgenic chicken, Non-transgenic chicken, *Bifidobacterium*, Growth traits

## Abstract

Lysozyme is one kind of antimicrobial proteins and often used as feed additive which can defend against pathogenic bacteria and enhance immune function of animals. In this study, we have injected the lentiviral vector expressing recombinant human lysozyme (rhLZ) gene into the blastoderm of chicken embryo to investigate the effect of recombinant human lysozyme on postnatal intestinal microbiota distribution and growth performance of chicken. Successfully, we generated 194 transgenic chickens identified by Southern blot with a positive transgenic rate of 24%. The average concentration of rhLZ was 29.90 ± 6.50 μg/mL in the egg white. Lysozyme in egg white of transgenic chickens had a significantly higher antibacterial activity than those of non-transgenic chickens by lysoplate assay (*P* < 0.05). The feces of transgenic and non-transgenic chickens were collected and five types of bacteria (*Lactobacillus, Salmonella, Bifidobacterium, Staphylococcus aureus and Escherichia coli*) were isolated and cultured to detect the impact of rhLZ on gut microbiota. Among the five bacteria, the number of *Bifidobacterium* in the intestine of those transgenic was significantly increased (*P* < 0.05). Moreover, the growth traits of the transgenic and non-transgenic chickens were analyzed. It was found that the 6-week shank length, 6-week weight and 18-week weight of transgenic chickens were significantly increased than that of non-transgenic chickens. The results demonstrated that rhLZ-transgenic chicken could promote the growth of *Bifidobacterium* in the intestine and improve the postnatal growth of chicken.

## Introduction

Lysozyme (EC.3.2.1.17) widely exists in eukaryotes and prokaryotes to protect organism from microbial invasion. Lysozyme functions by hydrolyzing β-1,4-glycosidic linkages between N-acetylglucosamine and N-acetylmuramic acid of the peptidoglycan layer in the bacterial cell wall (Chipman and Sharon 1969; Mai and Hu 2009). Lysozymes are generally classified into three major types: chicken type (c-type) (Kawamura et al. 2012), goose type (g-type) (Simpson et al. 1980) and invertebrate type (i-type) (Ito et al. 1999). The c-type lysozyme exists ubiquitously in many organisms including viruses, bacteria, plants, insects, fishes, reptiles, birds, and mammals (Maga et al. 2006a; Jolles 1996). Human lysozyme (hLZ), one of c-type lysozymes, is a positively charged molecule that consists of a polypeptide of 130 amino acid residues (Booth et al. 1997; Yu et al. 2013). It is widely distributed in body fluids, such as tears, saliva, and blood and plays an important role in host defenses (Jolles and Jolles 1984). Lysozyme in human milk can be passed to infants through breast feeding, which helps to establish infants’ innate immune barrier (Ellison and Giehl 1991; Nascimento and Giugliano 2000). Likewise, human lysozyme has been shown to have antifungal and antiviral activities (Kokoshis et al. 1978; Lee-Huang et al. 1999; Sava et al. 1988). Additionally, Lysozyme has a generally recognized as safe (GRAS) status for use in the food industry, owing to its no toxic effects when taken at levels of 5 mg/g body weight (Osserman et al. 1973). Ultimately, hLZ seems promising in food industries and therapeutic applications.

As the human body only secretes limited natural hLZ, several transgenic animals have been utilized to produce bioactive recombinant human lysozyme (rhLZ), including mice, goats, cows, and pigs (Cao et al. 2015; Lu et al. 2015; Maga et al. 2006b; Tong et al. 2011; Yang et al. 2011; Yu et al. 2006). rhLZ in milk has been demonstrated that it not only contributes to the lactating animal but also benefits to intestinal morphology, and modulates intestinal microbiota composition in infants to improve health of young animals (Brundige et al. 2008). In addition, rhLZ originated from transgenic animal milk is also of similar bioactivity as natural rhLZ (Liu et al. 2012), indicating that rhLZ expressed in animal production may replace natural hLZ in the future.

However, up to now, the expression of functional rhLZ in poultry is still rarely available. Therefore, producing rhLZ in egg white will effectively reduce the cost and present a promising application prospect. In this study, we generated transgenic chickens highly expressing rhLZ in egg white. The rhLZ exhibited similar physicochemical properties as natural hLZ, which revealed the potential of chicken eggs for hLZ producing.

## Materials and methods

### Construction of eukaryotic expression vector pHmLZ-IRES2 -EGFP and pChLZ-IRES2-EGFP

Human lysozyme fragment (accession number M19045) containing chicken signal peptide with *Bam*HI enzyme loci at 5 ‘end and *Xho*I enzyme loci at 3′ end was amplified from plasmid pGEM-T-HmLZ (gift from Prof Li Ning) using primers hLZ-F and hLZ-R (Table 1). The chicken lysozyme gene was amplified from cDNA of dwarf chicken (accession number X61198) using the primers ChLZ-F and ChLZ-R (Table 1). The two PCR products of human and chicken lysozyme fragments were ligated with pIRES2-EGFP (Sangon Biotech) after digested by *Xho*I and *Bam*HI, respectively, at the proportion of 3:1 under 16 °C over night. The recombinant vectors were transformed into DH5α competent cells.

**Table 1:** Primer list

Primes	Sequences
hLZ-F	5′-GCTTTGCTTCCTGCCCCTGGCTGCTCTGGGGAAGGTCTTTGAAAGGTGTGA-3′
hLZ-R	5′-CGCGGATCCCGGGCTCACACTCCACAACCTTGAACA-3′
ChLZ-F	5′-CCGCTCGAGGAATTCGCCACCATGAGGTCTTTGCTAATCTTGGTG-3′
ChLZ-R	5′-CGCGGATCCCGGGCTCACAGCCGGCAGCCTCTGA-3′
EGFP-F	5′-CAGTGCTTCAGCCGCTACCC-3′
EGFP-R	5′-TTCACCTTGATGCCGTTCTT-3′
ACTIN-F	5′-GAAACTACCTTCAACTCCATC-3′
ACTIN-R	5′-CGAGGCCAGGATGGAGCCGCC-3′
LYZ-F	5′-CGCCACCATGAGGTCTTTGC-3′
LYZ-R	5′-CGCGGATCCCGGGCTCACAC-3′
LYZ-F1	5′-CTAGCTAGCCGCCACCATGAGGTCTTTGC-3′
LYZ-R1	5′-CCGGAATTCTTACTTGTACAGCTCGTCCA-3′
I-F1	5′-TGTTATTTTCCACCATATTGCCGTC-3′
I-R1	5′-TCAGATCCCATACAATGGGGTACCT-3′
C-F1	5′-TACGGTAAACTGCCCACTTG-3′
C-R1	5′-GCGGCTATTGATCTGAAATA-3′
hLZ-S	5′-TGGGCGTGGATAGCGGTTTGA-3′
hLZ-A	5′-GGAATGCTCGTCAAGAAGACAGGGC-3′

### Transfection of 293FT cells with recombinant eukaryotic expression vectors

The approach of calcium phosphate transfection was used to transfect two recombinant expression vectors above and empty vector pIRES2-EGFP (1 μg/1μL) into 293FT cell (laboratory storage). Briefly, four transfection groups were designed: Control group: 293FT cells without transfection; Yp group: 293FT cells transfected with pIRES2-EGFP; Yh group: 293FT cells transfected with pHmLZ-IRES2 -EGFP; Yc group: 293FT cells transfected with pChLZ-IRES2-EGFP. 293FT cells were cultured in DMEM with 10% FBS at 5% CO_2_, saturated humidity, at 37 °C. Cells were transfected with four vectors above, respectively when cells reached 60–70% confluence. In order to detect transfection efficiency and expression of lysozyme, 293FT cells were collected at 48 h post transfection, and RNA was extracted for subsequent cDNA synthesis and fluorescence quantitative PCR using the primers LYZ-F, LYZ-R; EGFP-F, EGFP-R and ACTIN-F, ACTIN-R (Table 1). The expression level of EGFP was relative to ACTIN which acted as the reference gene.

### Antibacterial activity of 293FT cell supernatant

Since human and chicken lysozymes were secreted proteins, we detected antibacterial activity of human and chicken lysozyme against *Micrococcus* *lysodeikticus* ATCC no. 4698 (Sigma, S. Louis, MO, USA). A modification of Shugar’s turbidimetric method was used. Firstly, a standard calibration curve was generated with purified chicken egg lysozyme as follows: Lysozyme (0.01 g) was added into 1 mL PBS solution (pH 6.5), and then diluted 100 times to ensure lysozyme concentration up to 100 μg/mL. Six of twofold serial dilutions (3.12, 6.25, 12.5, 25, 50, 100 μg/mL) of lysozyme solution was made. The antibacterial activity of lysozyme in supernatant of 293FT cells was determined by suppression of lysozyme on *Micrococcus* *lysodeikticus*. Briefly, 5 mL of fresh *Micrococcus* *lysodeikticus* was centrifuged at 11,000*g* for 5 min, the supernatant was discarded and the pellet was washed twice with 50 mM sodium phosphate (pH 8.0). The initial measurement value was adjusted to OD = 0.5, either by adding more bacteria or buffer. The assay was started by the addition of supernatant of 293FT cells and the lysozyme absorbance was recorded every 30 s over 20 min using a spectrophotometer. One enzyme unit of enzyme activity corresponded to a decrease of 0.001 OD units each minute.

### Construction of lentivirus expression vector pLL3.7-HmLZ-IRES2-EGFP

HmLZ-IRES2-EGFP fragment with *Nhe*I enzyme loci at 5 ‘end and *Eco*RI enzyme loci at 3′ end was amplified from the recombinant eukaryotic expression vector pHmLZ-IRES2-EGFP using primers LYZ-F1 and LYZ-R1 (Table 1). PCR products were recycled and digested by *Nhe*I and *Eco*RI together with plasmid pLL3.7, then ligated at the proportion of 10:1 blending 16 °C for the night and transformed into DH5α competent cells. Recombinant plasmid pLL3.7-HmLZ-IRES2-EGFP positive clones were sequenced.

### Preparation and identification of transgenic chicken expressing rhLZ

Cell number of 293FT cells was adjusted to 1 × 10^5^/well and added to 24-well plates. Virus was centrifuged at 3000 rpm for 5 min, and then supernatant was added to the 293FT cells at a concentration of 1/100 or 1/10. Green fluorescent protein could be observed after 72 h. Virus titers was determined using the UltraRapid Lentiviral Titer Kit (System Biosciences, Mountain View, USA). 1 μL virus suspension at a virus titer of 1 × 10^6^ IU/mL was microinjected into the central part of the subgerminal cavity below the developing embryos of freshly laid eggs (stage-X) from dwarf chickens (Byun et al. 2013), then supplemented with suitable amount of egg white, sealed with paraffin wax and hatched normally.

PCR amplification was performed with 3 weeks old offspring using primers I-F1, I-R1 and C-F1, C-R1 (Table 1). Then Southern blot was used to identify the gene insertion. Briefly, about 20 μg DNA from chickens that PCR amplified positive were digested by *Hin*d III, and conducted slow electrophoresis under a low voltage about 40 V for 12 h. Southern blot analysis was implemented with specific hybridization probes which had been labelled with digoxigenin (DIG) using the PCR DIG Probe Synthesis Kit (Roche) after target fragments transferred to a Hybond-N membrane (Amersham Pharmacia Biotech, Piscataway, USA). Hybridization probes were amplified using the primers hLZ-S and hLZ-A (Table 1).

### rhLZ expression of transgenic chickens

An enzyme-linked immunosorbent assay (ELISA) was performed to monitor human lysozyme expression of transgenic chickens. Briefly, 10 μL serum of peripheral blood from positive rhLZ transgenic chickens of 20 weeks old was mixed with 990 μL sample diluent, then 100 μL mixed liquor was added to 96-well plate astest group, meanwhile, some wells were only added 100 μL sample diluent as control group, and 100 μL standard sample was added to other wells as standard group. Then a competitive inhibition reaction was launched between HRP labeled human lysozyme and unlabeled human lysozyme (standards or samples) with the pre-coated antibody specific for human lysozyme. OD_450_ absorbance was measured by microplate reader to calculate the sample concentration. ELISA kit could simultaneously detect the recombinant or natural lysozyme with no cross reaction with other related proteins.

### Antimicrobial property of rhLZ in egg white

Lysoplate assay was performed. Briefly, 1% agar plate containing 1 × 10^8^ CFU/mL of *Micrococcus lysodeikticus* sodium acetate buffer (0.1 M; pH 6.0) was prepared in petri dishes (approximately 5 mL per plate). When agar was solidified, wells were punched with the top of sterile pipette tips. Plates were refrigerated until use. A total of 20 μL PBS was added into each well as negative control, blank control was also set. Crude exact of egg white was dissolved in PBS and 20 μL was added to the wells of agar plate containing *Micrococcus lysodeikticus*. At last, the plates were incubated at 37 °C for 24 h and observed for bacterial growth or bacterial clearance.

### Phagocytic ability of monocyte from transgenic chickens

Monocyte plays an important role in both nonspecific and specific immune response, protecting the body from attacking by pathogenic microorganisms. In this study, a quick method (HCT8-MTT) was used to detect phagocytosis of monocyte/macrophage isolated from peripheral blood of fifteen individuals of transgenic chickens and non-transgenic chickens at the age of 90 days post hatch. Tumor cells treated by MTT were added to the mononuclear macrophage culture system, and then the ratio of OD_570_ of tumor cells swallowed and adhered to control cells was defined as phagocytosis product.

### Distribution of microbial flora in feces between transgenic and non-transgenic chickens

About 1 g fresh feces were collected in the sterile tubes weighed in advance from fifteen individuals of transgenic chickens and non-transgenic chickens at the age of 35 weeks post-hatch respectively, and then put into the anaerobic tank immediately, followed with bacteria isolation and culture. Finally, the number of *Lactobacillus*, *Salmonella*, *Bifidobacterium*, *Staphylococcus aureus* and *Escherichia coli* was detected.

### Detection of growth performance between transgenic and non-transgenic chickens

Transgenic chickens and non-transgenic chickens were raised in the same chamber with commercial feed ad libitum. Growth traits including birth weight, 6-week shank length, 6-week weight, 18-week weight of transgenic and non-transgenic chickens were recorded and analyzed.

### Statistical analysis

Data are shown as mean ± SEM (lysozyme assay). All data was analyzed by a one-way Analysis of Variance (ANOVA) followed by Tukey’s post hoc test (software: SPSS 16.0 for Windows, SPSS Inc., USA).

## Results

### Transfection efficiency of eukaryotic expression vectors and expression of EGFP in transfected 293FT cells

**Fig. 1:**
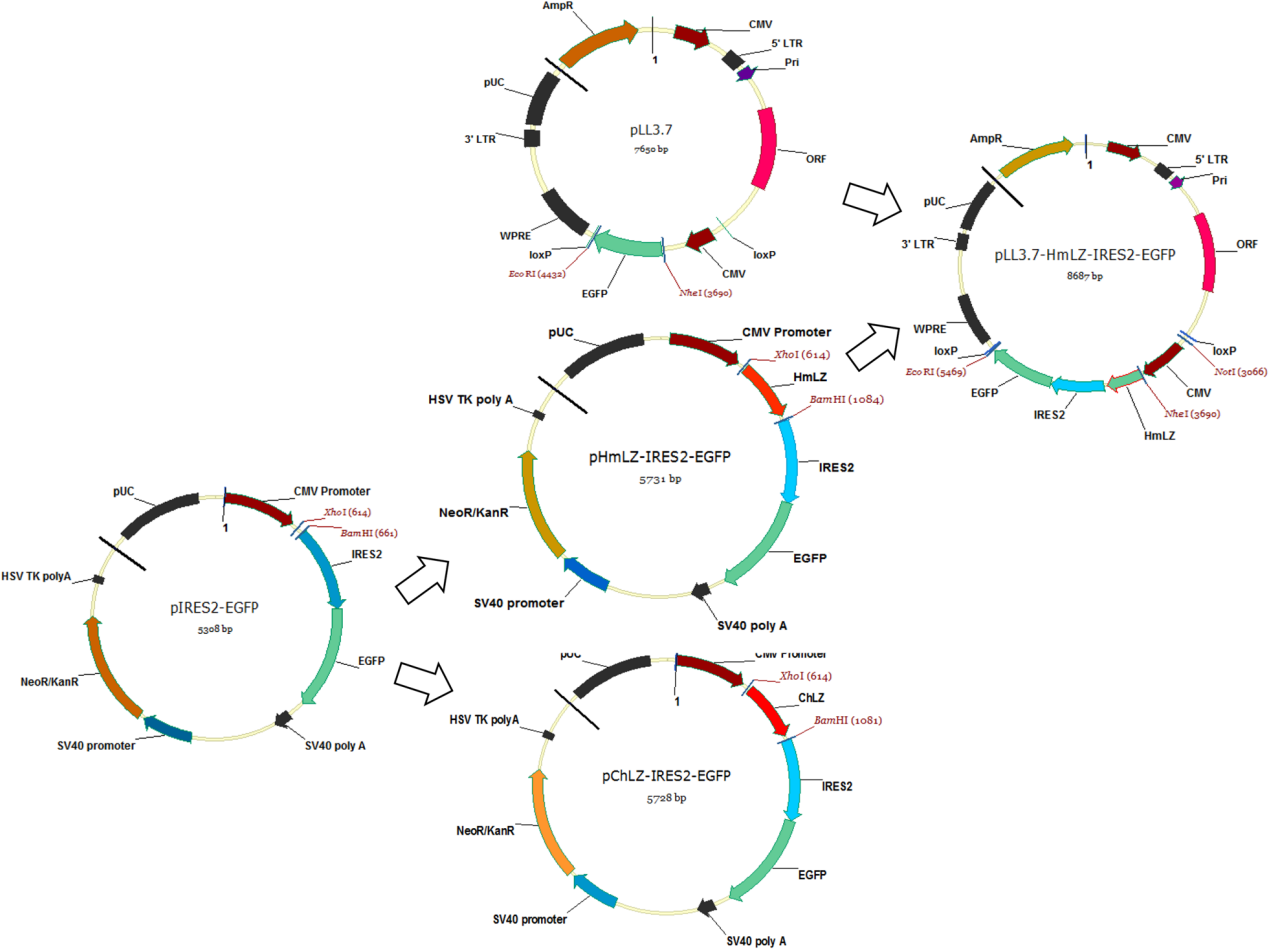
Schematic diagram for eukaryotic expression vectors pChLZ-IRES2-EGFP and pHmLZ-IRES2-EGFP and lentivirus expression vector pLL3.7-HmLZ-IRES2-EGFP

**Fig. 2:**
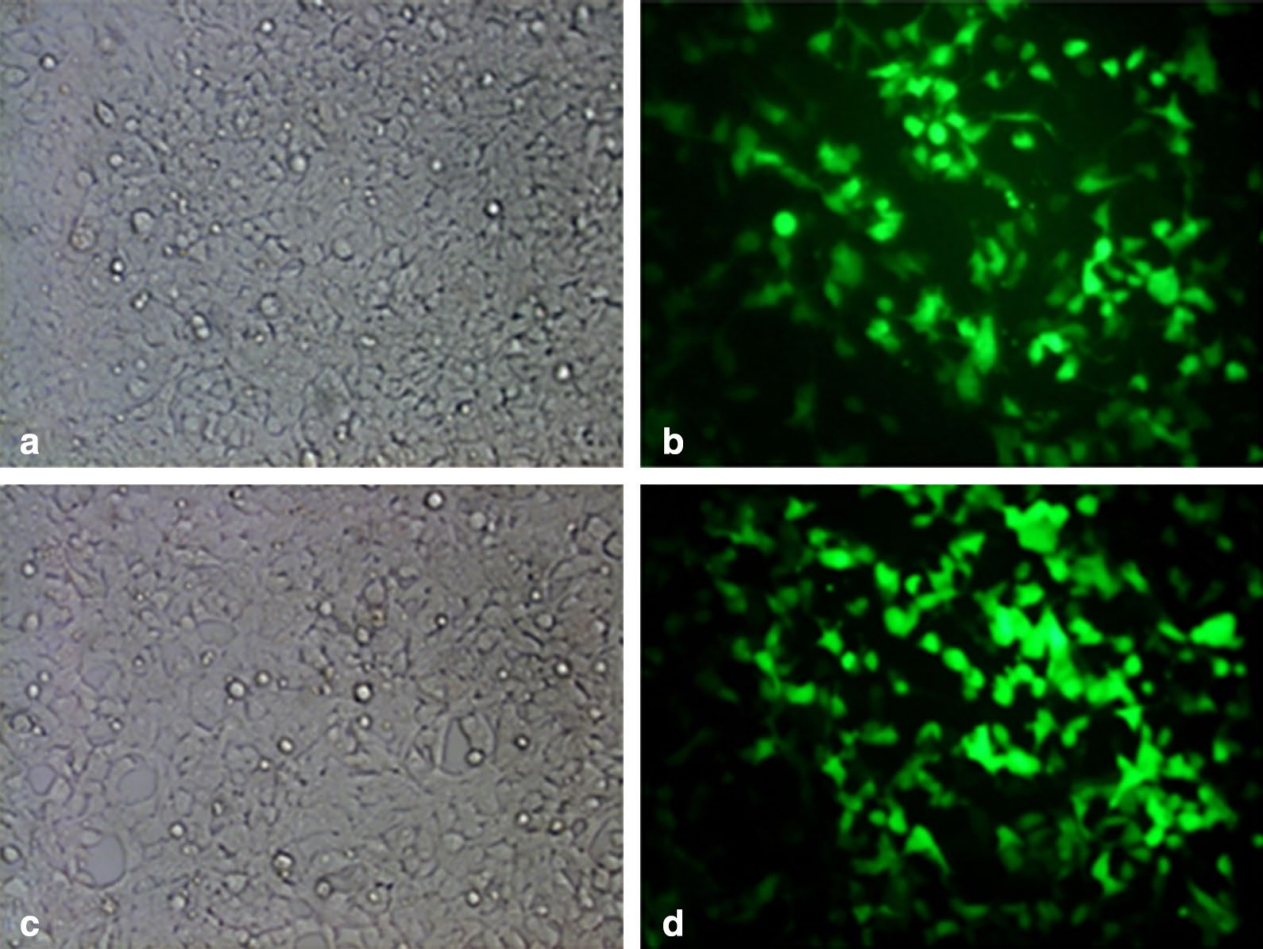
The transient expression result of vectors in 293FT cell line. **a** and **b** Represent the transient expression of pIRES2-HMLZ-EGFP in 293FT cell line (×100). **c** and **d** Represent the transient expression of pIRES2-CHLZ-EGFP in 293FT cell line (×100). Besides, **a** and **c** showed the fluorescent background, while **b** and **d** showed the EGFP observation

Eukaryotic expression vectors pChLZ-IRES2-EGFP and pHmLZ-IRES2-EGFP were constructed (Fig. 1). 293FT cells transfected with pChLZ-IRES2-EGFP showed highest expression of green fluorescent protein at 48 h post transfection (Fig. 2a, b), 293FT cells transfected with pHmLZ-IRES2-EGFP showed medium expression (Fig. 2c, d), and 293FT cells transfected with pIRES2-EGFP showed low expression (data not shown). Results showed that it was proportional to cell transfection efficiency.

**Fig. 3:**
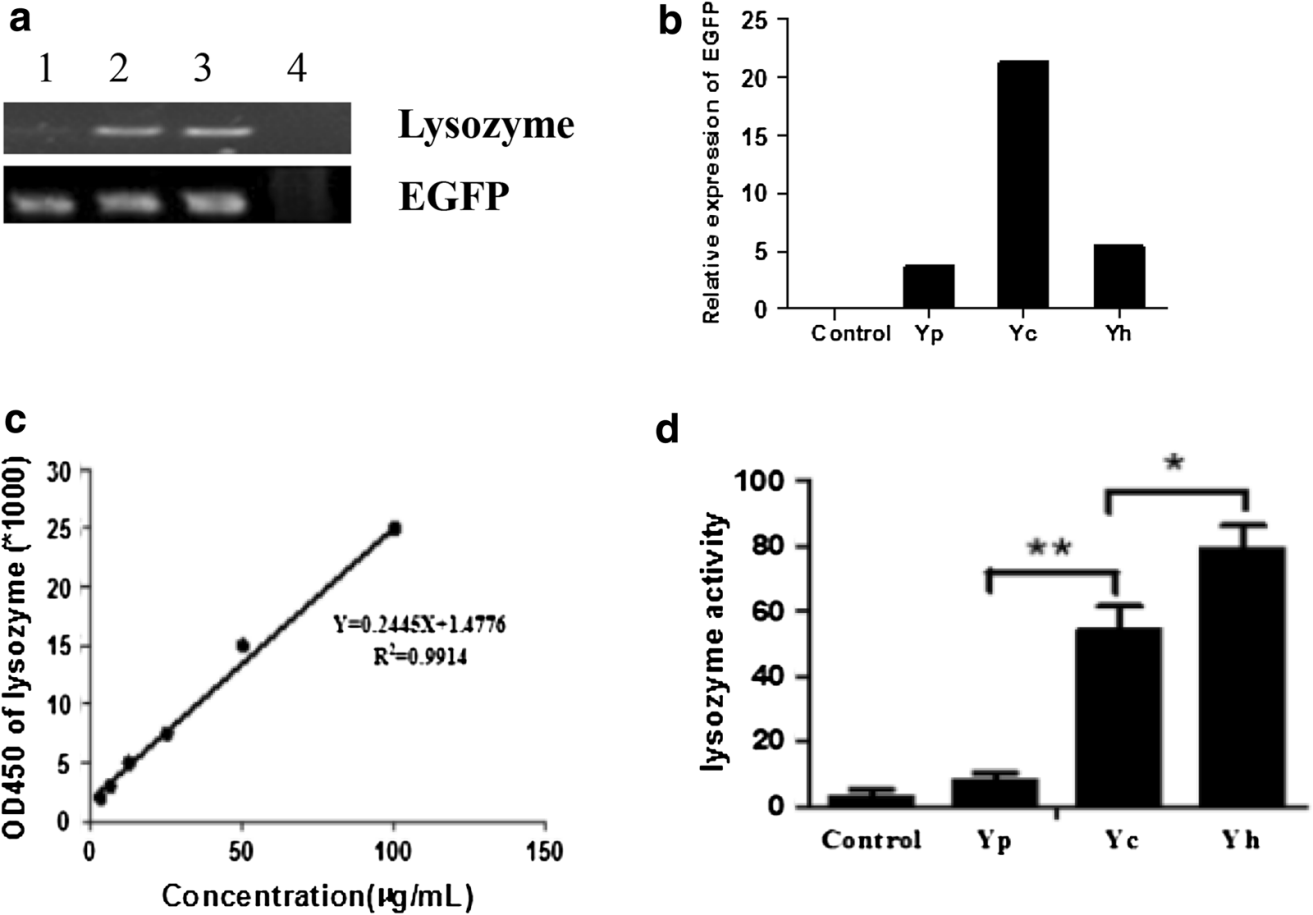
Relative expression of EGFP in transfected 293FT cells and antibacterial activity of supernatant of 293FT cells. **a** PCR amplification of lysozyme and EGFP in transfected 293FT cells. *Lane 1*, *2*, *3*, and *4* shows expression of lysozyme and EGFP in Yp group, Yh group, Yc group and control group, respectively. **b** qPCR detection of EGFP expression in transfected 293FT cells. **c** Standard curve of lysozyme activity. **d** Antibacterial activity against micrococcus lysodeikticus of supernatant of 293FT cell. Control group: 293FT cells without transfection; Yp group: 293FT cells transfected with pIRES2-EGFP; Yh group: 293FT cells transfected with pHmLZ-IRES2-EGFP; Yc group: 293FT cells transfected with pChLZ-IRES2-EGFP. Statistical significance is presented as follows: *P < 0.05; **P < 0.01

Gel electrophoresis showed that lysozyme was expressed in both 293FT cells transfected with pHmLZ-IRES2-EGFP and pChLZ-IRES2-EGFP. The 293FT cells transfected with pIRES2-EGFP appeared no expression of lysozyme; Green fluorescent protein did not express in control group (Fig. 3a). In addition, Quantitative PCR results showed that expression of EGFP in Yp, Yc and Yh group was 3.6, 21.3, and 5.4, respectively (Fig. 3b). Expression of green fluorescent protein in group Yc was four times of that of group Yh; therefore, transfection efficiency of group Yc was four times of group Yh. Since expression of proteins on both sides of IRES2 regulatory elements was the same, then we could speculate that amount of chicken lysozyme expression was four times of human lysozyme after 48 h transfection.

### Detection of antibacterial activity in supernatant of 293FT cells

Compared to the control group, Yp group represented no significant difference in antibacterial activity against *Micrococcus lysodeikticus*. Both of them showed no obvious antibacterial effect (Fig. 3d). Yc and Yh group showed stronger antibacterial activity than Yp group (*P* < 0.01). Yh group showed stronger antibacterial activity than Yc group (*P* < 0.05). In addition, average enzyme activity of egg white lysozyme was 54 U in Yc group, 79 U in Yh group. Antibacterial activity against *Micrococcus lysodeikticus* of recombinant human lysozyme was about six times than that of chicken lysozyme.

### Observation of EGFP in 293FT cells infected with lentivirus

**Fig. 4:**
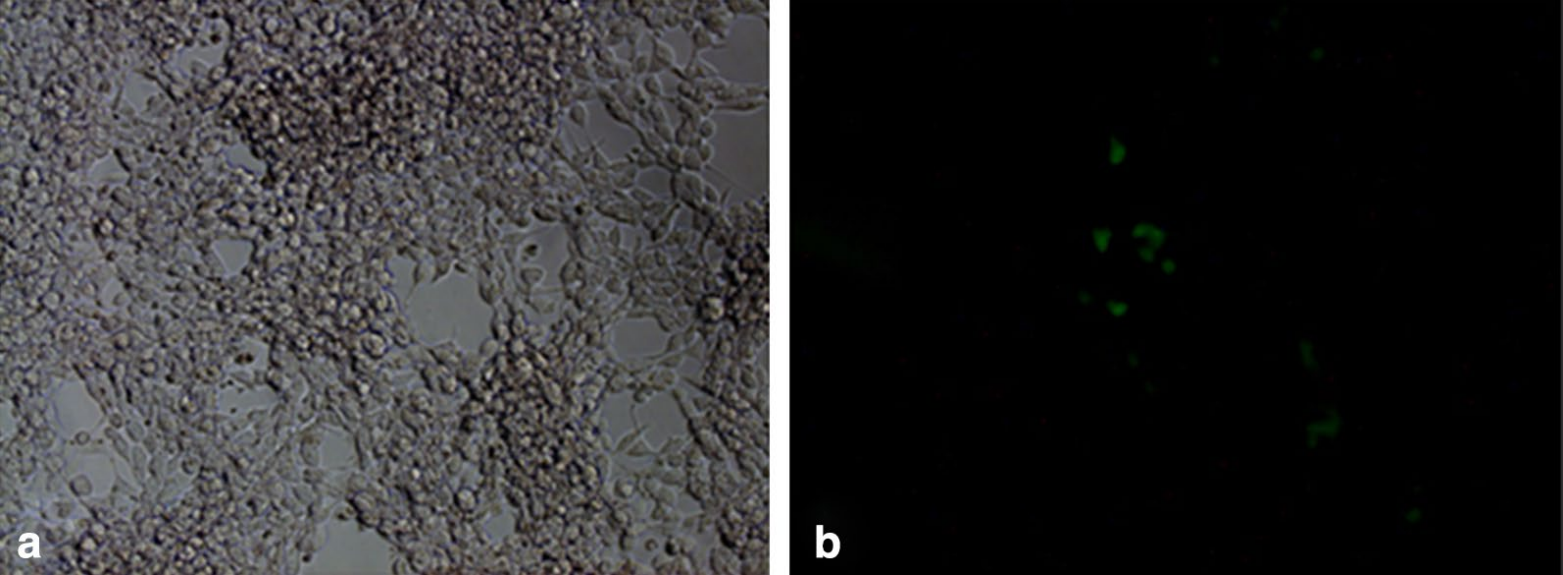
The expression of EGFP in 293FT cells at 72 h post infection (100X). **a** Represents the fluorescent background; **b** represents EGFP observation

The expression of green fluorescent protein in 293FT cells infected with lentivirus could be observed under microscope at 72 h post transfection (Fig. 4). As calculated, virus titer was 1 × 10^6^ TU/mL.

### Identification of transgenic chickens

**Fig. 5:**
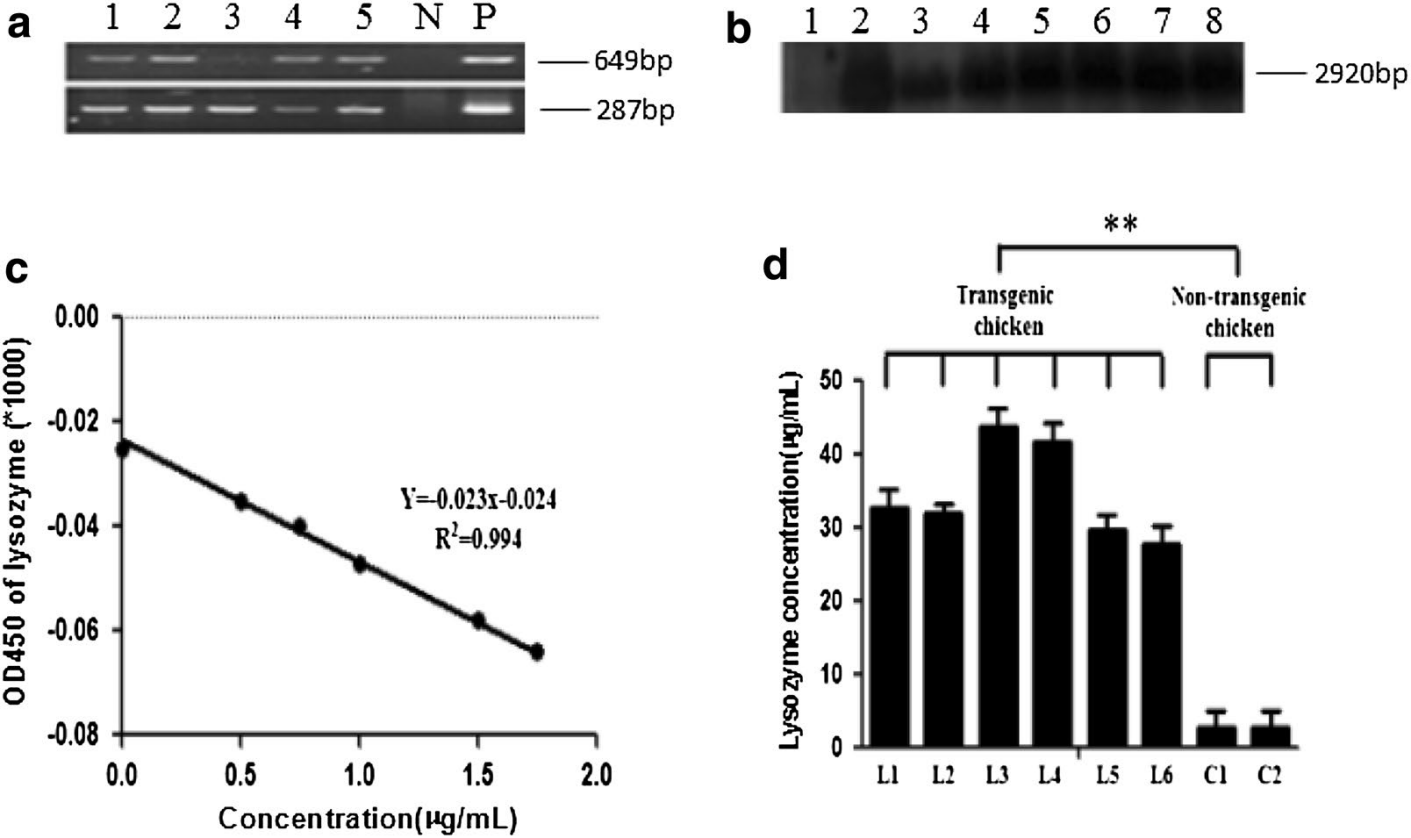
Preparation, identification and rhLZ expression of transgenic chickens **a** PCR amplification of the exogenous rhLZ in transgenic chicken, samples from *lane 1* to *lane 5*, *lane P* (positive plasmid) and *lane N* (negative control) were listed; **b** Detection of rhLZ in transgenic chickens by *Southern blot*. Samples from *lane 2* to *lane 8* showed for the positive transgenic chickens, while sample of *lane 1* represented non-transgenic chicken. **c** Standard curve of rhLZ expressed in the serum. **d** Concentration of rhLZ in the serum of both transgenic and non-transgenic chickens

The lentivirus expression vector pLL3.7-HMLZ-IRES2-EGFP (Fig. 1) was constructed and used to prepare transgenic chickens. DNA from peripheral blood of transgenic chickens was amplified, followed with detection of Southern blot. 649 bp PCR products and 287 bp PCR products were obtained indicating that human lysozyme gene might have been integrated into chicken genome (Fig. 5a). Totally, 194 transgenic chickens were identified by Southern blot with a positive transgenic rate of 24% (Fig. 5b).

### Detection of rhLZ expression of transgenic chickens

OD_450_ of transgenic chicken, wild chicken and standard samples were read. According to the formula of y = −0.023 × −0.024(R^2^ = 0.994) (Fig. 5c), we could calculate the concentration of lysozyme in serum. Lysozyme concentration of six transgenic chickens was 32.9, 32.0, 43.7, 41.8, 29.9, and 27.9 μg/mL, respectively. The lysozyme for two non-transgenic chickens was 2.8 and 2.7 μg/mL, respectively. Average lysozyme concentration of transgenic chicken was 29.9 μg/mL (Fig. 5d). Since similar structure between human and chicken lysozyme, they couldn’t be detected separately. The concentration here was combination of human and chicken lysozyme. It is obvious that lysozyme concentration in serum of transgenic chicken was ten times of those non-transgenic.

### Antimicrobial property of rhLZ in egg white tested by agar diffusion test

**Fig. 6:**
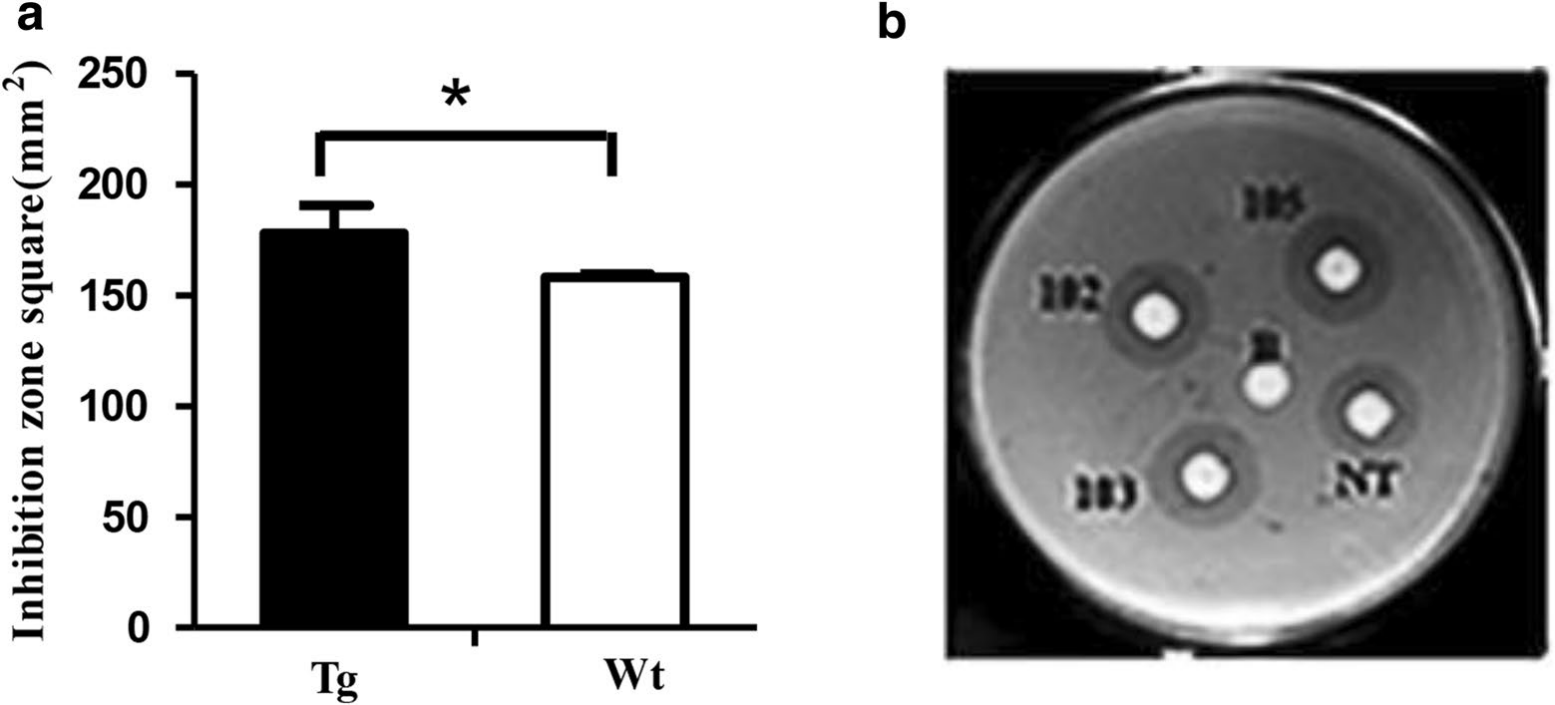
Antimicrobial property of rhLZ in egg white tested by agar diffusion test. **a** The inhibition zone square of the transgenic chickens and non-transgenic chickens. **b** *Diagrammatic sketch* of the agar diffusion test for the transgenic group and non-transgenic group. Individuals of *102*,*103* and *105* represented transgenic chickens, NT represented non-transgenic chicken; B represented blank

Egg white of transgenic chickens named 102,103 and 105 were collected. Simultaneously, egg white of age-matched non-transgenic chicken named NT was also gathered. After adsorbing of different sample filters, solid medium would appear bacterial plaques of different sizes, with no plaques presenting in blank. Antibacterial activity was measured by the size of bacteriostatic circle. The results showed that transgenic chickens expressed significantly higher antimicrobial property than that of non-transgenic chickens (*P* < 0.05) (Fig. 6).

### Phagocytic ability of monocyte from transgenic and non-transgenic chickens

**Fig. 7:**
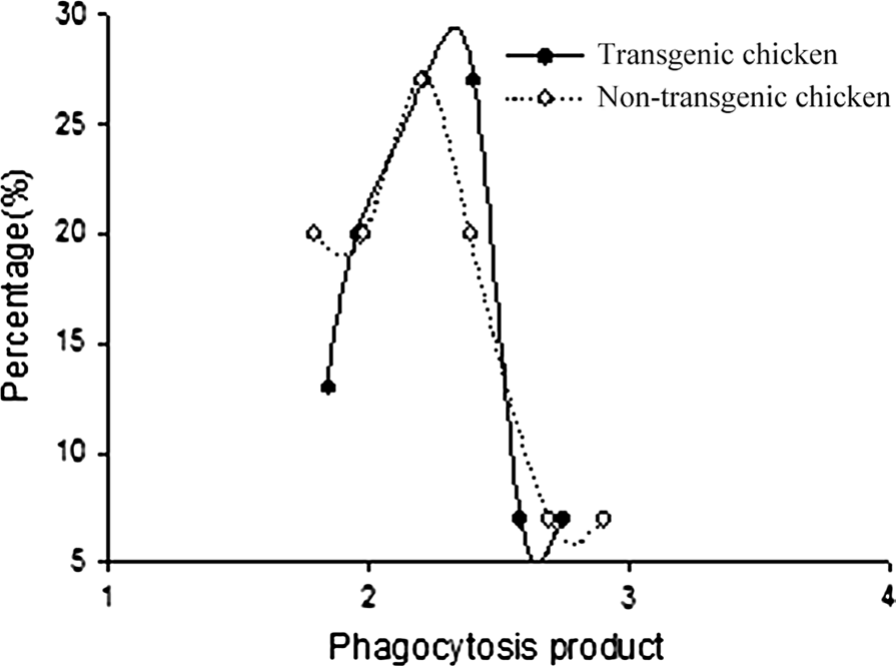
Phagocytic ability of monocyte from transgenic chickens and non-transgenic chickens. *X axis* represents phagocytosis product; *Y axis* represents percentage of transgenic chickens and non-transgenic chickens

It was showed that the phagocytosis products were between 1.5 and 3.0 (Fig. 7), and normally distributed. Compared with non-transgenic chickens, monocyte phagocytosis of transgenic chickens tended a bit stronger and did a same population trend.

### Difference of microbiota in feces between transgenic chickens and non-transgenic chickens

**Fig. 8:**
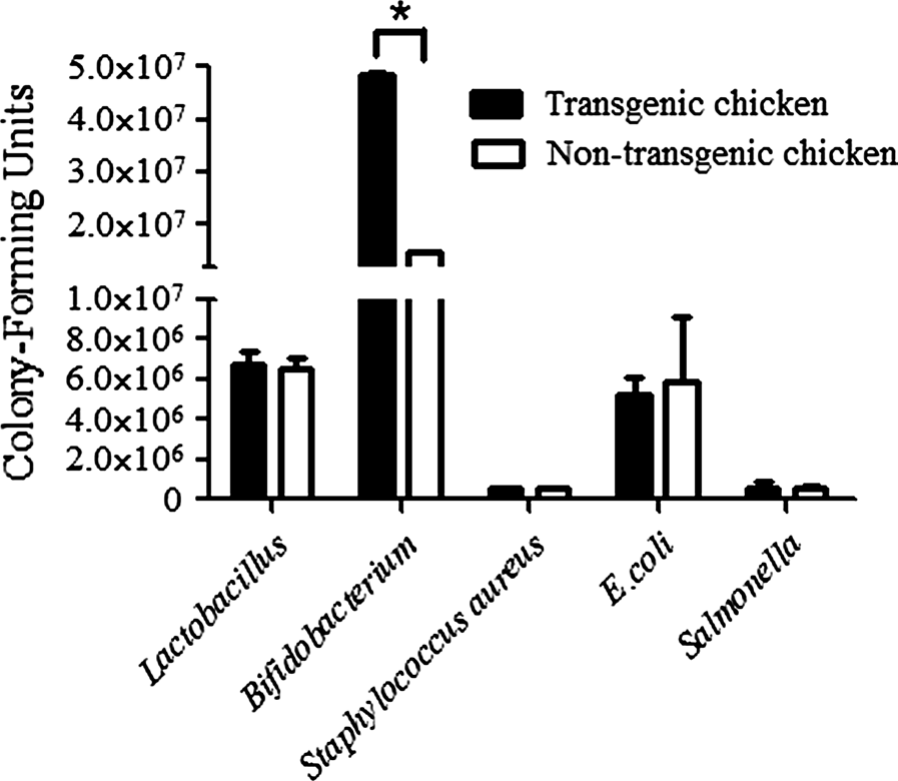
Distribution of *Lactobacillus, Bifidobacterium, Staphylococcus aureus, Escherich.coli* and *Salmonella* in the feces of transgenic chickens and non-transgenic chickens. An *asterisk* [*] marked in graph indicates statistical significance

Five types of bacteria were separated and cultured to analyze the impact of rhLZ on gut microbiota. The number of *Bifidobacterium* in the feces of transgenic chickens was significantly increased (4.83 × 10^7^ ± 0.66 × 10^6^ CFU vs 1.46 × 10^7^ ± 0.13 × 10^6^ CFU, *P* < 0.05). The number of *Staphylococcus aureus*, *Escherich.coli*, *Salmonella*, and *Lactobacillus* appeared no significant difference (Fig. 8).

### Analysis of growth performance of transgenic chickens and non-transgenic chickens

**Fig. 9:**
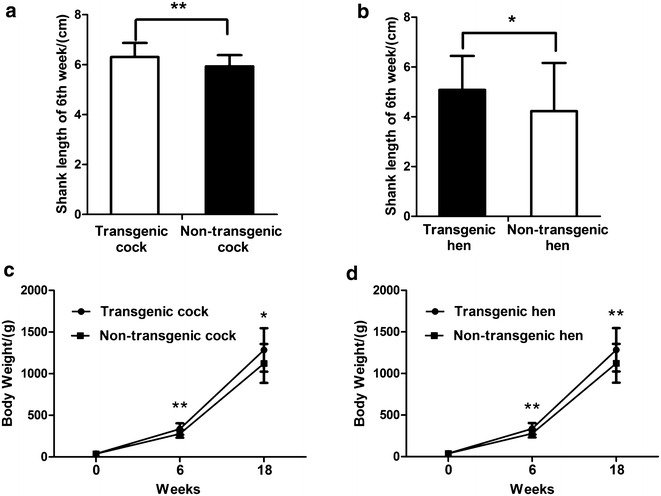
Variation of 6-week shank length and body weight variation of transgenic chickens and non-transgenic chickens. **a** and **b** reflect difference of 6-week shank length between those transgenic and non-transgenic; **c** and **d** indicate body weight varied at born, 6 and 18 weeks

Growth traits of transgenic chickens (100 individuals) and non-transgenic chickens (100 individuals) were collected. It was found that the 6-week shank length, 6-week weight and 18-week weight of those transgenic were significantly increased (Fig. 9).

## Discussion

As lentiviral vector can carry large exogenous gene (~8 kb) and integrate these genes into the genomes of both dividing and non-dividing cells, it is considered to be an ideal candidate for transporting genetic material into cells and tissues (Harvey et al. 2002a, b; McGrew et al. 2004; Sang 1994; Semple-Rowland and Berry 2014). Lentiviral vector systems have been considered to be the most successful method for generating transgenic chickens through delivering genes to embryos in newly laid eggs (Byun et al. 2013; Kwon et al. 2004; Lillico et al. 2007). The stable transmission of the integrated vector through the germ line was demonstrated with conserved expression of exogenous gene in the G1 and G2 generations (Byun et al. 2011).

Due to its inherent antimicrobial activity, human lysozyme (hLZ) in egg white has the potential to improve the nutritional value and antibacterial activity of egg. Researchers reported that hLZ expressed in the mammary gland in transgenic mice enhanced the antimicrobial properties of milk (Maga et al. 1998). hLZ is more bioactive than its avian counterparts (White et al. 1988), thereby adding hLZ to chicken via genetic engineering will help to increase the levels of endogenous lysozyme. Consistent to these findings, we found that egg white of transgenic chickens exhibited a stronger antibacterial activity by agar diffusion test in this study. Reports showed that phagocytosis of monocytes-macrophages was a marker for disease resistance of dwarf chickens (Ma et al. 2010). In this study, monocytes-macrophages phagocytosis of transgenic chickens tended a bit stronger than non-transgenic chickens, which probably indicated that hLZ could strengthen chickens, disease resistance. hLZ is normally present in the tears, saliva, and egg white of chicken without causing any allergic response when consumed by humans or animals. However, it usually brings about a problem that rhLZ may be expressed in a form of inclusion, only exhibiting its activity after refolded. In this study, results of the Elisa analysis showed that transgenic chickens expressing rhLZ using a lentiviral vector system was successful. Moreover, previous research revealed that rhLZ overexpression in rabbit caused a lactation problem (Houdebine 2009); however, there were no difficulties in producing the next generation of these transgenic chickens.

Considering the impact factors that lentiviral vector systems can deliver exogenous DNA fragments of approximately 8 kb, viral titers are inversely proportional to the size of inserted fragments and virus packaging efficiency, a maximum size of the 5′-regulatory region of the CMV gene that can be packaged into virus particles was selected. Finally, the plasmid pLL3.7-HmLZ-IRES2-EGFP composed of the CMV promoter of 553 bp and hLZ gene of 447 bp was constructed and used to produce the recombinant virus particles.

In order to realize rhLZ secreted into chicken cells, human lysozyme signal peptide was replaced by chicken lysozyme signal peptide. It was confirmed that chicken signal peptide could direct rhLZ secretion into chickens. In this study, we obtained transgenic chickens expressing bioactive human lysozyme to an amount of 29.9 μg/mL with excluded gene silence and inhibition that usually happen in transgenic chickens prepared by lentiviral vector (Mizuarai et al. 2001). The expression levels of rhLZ were different among individuals, probably resulting from position effects caused by chromosomal location of the transgene insertion (Dobie et al. 1996; Kong et al. 2009; Robertson et al. 1995). Therefore, selecting of transgenic chickens expressing rhLZ of high levels will be available for commercial production.

In feeding trials using animal models, pasteurized milk from HLZ transgenic animals was capable of modulating microbial growth in the intestine (Maga et al. 2006c). Lysozyme is thought to help contribute to a healthier gastrointestinal tract (Goldman 2007; Lonnerdal 2003). So we detect the intestine flora of both transgenic chickens and non-transgenic chickens by analyzing their feces collected. In our study, the number of *Bifidobacteria* of those transgenic was significantly higher than those non-transgenic indicating that rhLZ promotes *Bifidobacteria* proliferation. *Bifidobacteria*, normal habitants of the intestinal tract amounts to 10^10^ CFU/g of intestinal contents (Tannock 1995). These bacteria exhibit several healthy, nutritional and therapeutic benefits to hosts including reduction of blood cholesterol (Modler 1994), improvement of lactose utilization in malabsorbers (Tianan Jiang Amad 1996), and increased immunity in animal hosts (Ali 2009; Gill 1998). They are believed to be essential for maintaining the healthy equilibrium between beneficial and potentially harmful microorganisms in the gastrointestinal tract (Sreekumar and Hosono 1998). In terms of clinical reports, *Bifidobacteria* tend to reduce the incidence of rotaviral infection, traveller’s diarrhea and antibiotic associated diarrhea (Zuccotti et al. 2008). They also have effects on pathogens belonging to the genera *Salmonella*, *Escherichia*, *Proteus*, *Shigellaand Candida* (Dicks and Botes 2010). Although, lysozymes are reportedly more effective against gram-positive bacteria lacking the outer membrane found in gram-negative bacteria (Costerton et al. 1974). Surprisingly, *Bifidobacterium* appeared to be more resistant than other Gram positive bacteria and Gram negative bacteria to lysozyme in the current study.

Additionally, we found that transgenic chickens showed significantly high growth performance than non-transgenic chickens, but of similar hatching rate and healthy chick rate (data not shown). It might indicate that rhLZ did not affect hatch and health of chickens. Furthermore, exogenous gene, rhLZ, improved the growth of postnatal chickens by modulating their intestine flora structure thereby promoting intestine immune and facilitating chicken growth and development.

With the result that *Bifidobacteria* appear increased in recombinant human lysozyme transgenic chickens and these transgenic chickens gained more weight at postnatal, Interest comes that whether *Bifidobacteria* has a direct or indirect correlation with lysozyme.

In conclusion, we demonstrate that lentivirus-mediated expression of recombinant human lysozyme in transgenic chickens is feasible. The lysozyme concentration is much higher in transgenic chicken than that in non-transgenic chicken, which indicated that albumen from transgenic chicken showed a higher antibacterial activity. rhLZ-transgenic chicken promote *Bifidobacteria* proliferation and improve the growth of postnatal chicken.

## Abbreviations

PCR: polymerase chain reaction
DMEM: Dulbecco’s modified eagle medium
FBS: fetal bovine serum
DNA: desoxyribonucleic acid
cDNA: copy DNA
CMV: cytomegalovirus
IRES: internal ribosome entry site
EGFP: enhanced green fluorescent protein
ATCC: American type culture collection
PBS: phosphate-buffered saline
OD: optical density
bp: base pair(s)
ELISA: enzyme-linked immuno sorbent assay
MTT: 3-(4,5-Dimethylthiazol-2-yl)-2,5-diphenyltetrazolium bromide
CFU: colony-forming unit
Kb: kilo base pair(s)
ng: nanogram
LB: Luria-Bertoni
RNA: ribonucleic acid
min: minute(s)
h: hour(s)
µg: microgram
